# Association between serum uric acid and prediabetes in a normal Chinese population: A cross-sectional study

**DOI:** 10.1097/MD.0000000000040544

**Published:** 2024-11-29

**Authors:** Keqing Shen, Yilin Huang, Junlu Zhang, Liangli Chen, Xixuan Cai, Jianjiang Pan, Jingyi Li, Lusha Li, Liying Chen

**Affiliations:** a Department of General Practice, Sir Run Run Shaw Hospital, School of Medicine, Zhejiang University, Hangzhou, Zhejiang, China; b Department of Pathology, Second Affiliated Hospital of Wenzhou Medical University, Wenzhou, Zhejiang, China.

**Keywords:** diabetes, gender differences, prediabetes, serum uric acid

## Abstract

Cardiovascular events are frequent among individuals with prediabetes. And the relationship between cardiovascular diseases and elevated serum uric acid (SUA) levels has been supported by extensive scientific evidence. However, there remains controversy regarding the correlation between elevated SUA and prediabetes. The aim of this study was to investigate the association between elevated SUA levels and the prevalence of prediabetes and gender differences in the association. A total of 190,891 individuals who participated in health checkups at the Health Promotion Center of Sir Run Run Shaw Hospital of Zhejiang University from January 2017 to December 2021 were included in this cross-sectional study. The health checkups were carried out by trained general practitioners and nurses. The diagnostic criteria for diabetes and prediabetes are defined in the Standards of Medical Care in Diabetes-2022. The association between SUA levels and diabetes and prediabetes was examined based on logistic regression analysis. The dose-response effect between SUA levels and diabetes and prediabetes in both sexes was assessed using a restricted cubic spline (RCS) regression model. Among 190,891 participants, this study included 106,482 males (55.8%) and 84,409 females (44.2%). There were 46,240 (24.2%) patients with prediabetes and 20,792 (10.9%) patients with diabetes. SUA was divided into quartiles (Q). Compared to the SUA Q1 group, the prevalence of prediabetes was elevated in the SUA Q4 group (OR = 1.378, 95% CI = 1.321–1.437), but diabetes risk was decreased in the SUA Q4 group (OR = 0.690, 95% CI = 0.651–0.730). We found that SUA levels were correlated with prediabetes more significantly in male subjects (OR = 1.328, 95% CI = 1.272–1.386) than in female subjects (OR = 1.184, 95% CI = 1.122–1.249) (*P* for interaction < .001). Higher SUA levels were strongly related to an elevated prevalence of prediabetes but a decreased prevalence of diabetes. The association of SUA in prediabetes was more significant in men.

## 1. Introduction

The prevalence of a high-calorie, high-sugar diet and the demands of sedentary work have contributed to a rise in the incidence of diabetes, which has been posing a growing health crisis around the world. From the International Diabetes Federation’s Diabetes Atlas, it has been revealed that the global percentage of diabetes has reached 10.5% in 2021.^[1]^ Prediabetes is an intermediate-glycaemic state between normoglycaemia and type 2 diabetes, which has abnormal carbohydrate metabolism. Prediabetes includes impaired fasting glucose and abnormal glucose tolerance.^[2,3]^ The prevalence of prediabetes is gradually increasing, and it is becoming more common than diabetes. According to the most recent report of China, the prevalence of diabetes rose from 10.9% in 2013 to 12.4% in 2018, with the prevalence of prediabetes also increasing from 35.7% in 2013 to 38.1% in 2018.^[4]^ The United States (US) Preventive Services Task Force Recommendation Statement indicated that an estimated 34.5% of all US adults meet criteria for prediabetes in 2021.^[5]^ Studies showed that about 5.8% to 18.3% of people with prediabetes progress to diabetes each year.^[6–9]^ In addition, cardiovascular events are significantly more likely to develop in people with prediabetes.^[10–12]^ Therefore, early identification of people with prediabetes and lifestyle modifications are crucial for reducing the risk of developing diabetes and cardiovascular disease.

Serum uric acid (SUA) is the final product of purine catabolism in the human body. Excessive production of SUA or reduced excretion can both lead to hyperuricemia. If left uncontrolled, hyperuricemia can progress to gout.^[13]^ The link between SUA and various cardiovascular diseases is well supported by extensive scientific studies, including hypertension, metabolic syndrome, coronary artery disease, and cerebrovascular disease.^[14,15]^ A study of the Third National Health and Nutrition Examination Survey showed that SUA are independently associated with total and cardiovascular mortality.^[16]^ A multicenter cohort study reported that SUA levels were significant and independent risk factors for cardiovascular disease mortality.^[17]^

Several studies have shown that a high SUA level is significantly associated with diabetes, but the association remains controversial. Some of the studies recorded a positive association between the two,^[18–23]^ while others found a negative relationship.^[24–26]^ There was also a study that reported no association.^[27]^ Moreover, studies on sex-specific associations between SUA and diabetes have been conflicting. A Chinese hypertensive population study found that higher uric acid levels were significantly associated with an increased risk of new-onset diabetes in women but not men.^[19]^ A cohort study in Sweden found that higher SUA levels were related to an elevated incidence of diabetes and were more significant among men than women.^[28]^ A study in the US population found a positive correlation between prediabetes risk and SUA that was stronger in women than in men, while a negative association between diabetes and SUA was stronger in men than in women.^[29]^

Previous research on the relationship between SUA and different health outcomes has mostly concentrated on individuals with specific diseases such as diabetes or hypertension. Studies on the link between SUA and the incidence of prediabetes are limited and usually have relatively small sample sizes. Based on previous research, the aim of this research was to examine the association between different SUA levels and prediabetes. In addition, this study investigated the impact of SUA levels on prediabetes and diabetes, with specific attention to gender disparities. This study hypothesized that high SUA levels increased the prevalence of prediabetes.

## 2. Methods

### 2.1. Study population

The subjects of this cross-sectional study were composed of individuals who chose to participate in health checkups conducted at the Health Promotion Center of Sir Run Run Shaw Hospital, Zhejiang University School of Medicine, from January 2017 to December 2021. The inclusion criteria were participants in the age range 18 to 80 years old (n = 386,992). The exclusion criteria were participants with missing past history, smoking history, drinking history (n = 26,075) or those with missing information on waist measurement, height, weight, and metabolic factors (n = 65,870) or those who had repeat checkups (n = 104,156). Finally, 190,891 subjects were analyzed. The sample size of this cross-sectional study was estimated by combining the prevalence of prediabetes in the Chinese population with a confidence level of 0.95 and a confidence interval width of 0.04.

The study was conducted in accordance with the Declaration of Helsinki and approved by the Ethics Committee of Sir Run Run Shaw Hospital, affiliated with Medical College of Zhejiang University (No. 2023-0562). Patient consent was waived due to the research using the data obtained in the previous clinical diagnosis and treatment, without using the medical records that the patient has clearly refused to use. The study will not adversely affect the rights and health of the subjects, and the privacy and personal identity of the subjects will be protected.

### 2.2. Data collection

In the physical examination, trained general practitioners were responsible for collecting the gender, age, past medical history, smoking history, and drinking history of the subjects through face-to-face interactions. Trained nurses utilized calibrated standard instruments to measure the height, weight, waist circumference (WC), systolic blood pressure (SBP), and diastolic blood pressure (DBP) of each individual. Overnight rapid venous blood samples from the subjects were collected, including total cholesterol (TC), triglyceride (TG), fasting plasma glucose (FPG), glycosylated hemoglobin (HbA1_C_), blood urea nitrogen (BUN), creatinine (Cr), high-density lipoprotein cholesterol (HDL-C), low-density lipoprotein cholesterol (LDL-C) and SUA. Using the formula of weight divided by squared height, the body mass index (BMI) was calculated.

### 2.3. Assessment of variables

The diagnostic criteria of diabetes and prediabetes were defined in the Standards of Medical Care in Diabetes-2022 of the American Diabetes Association.^[3]^ Diabetes was defined as HbA1_C_ ≥ 6.5%, or FPG ≥ 7.0 mmol/L, or a self-reported diabetes diagnosis. Prediabetes was defined as HbA1_C_ 5.7% to 6.4% or FPG between 5.6 to 6.9 mmol/L, without a self-reported diagnosis of diabetes. Normoglycaemia was identified on the basis of FPG < 5.6 mmol/L and not satisfying the diagnostic criteria for prediabetes and diabetes.

On the basis of the quartiles (Q) of SUA distribution, 4 groups of uric acid were formed (≤282 μmol/L, 282–344 μmol/L, 344–411 μmol/L, and >411 μmol/L). This study collected information on sex (male and female), smoking history (current and no), and drinking history (current and no). Past history, including diabetes, hypertension and hyperlipidemia, was also categorized into yes and no. Other covariates, including age, SBP, DBP, BMI, WC, TC, TG, BUN, Cr, HDL-C, and LDL-C, were analyzed as continuous variables in this study.

### 2.4. Statistical analysis

The statistical analyses for this study were conducted using SPSS version 25.0 and R version 4.2.3. In the statistical analysis of this study, quantitative variables are presented as the means ± standard deviations or medians (interquartile ranges), and ANOVA was applied to 3 or more groups. Qualitative variables are presented as percentages, and the Chi-square test was applied to identify significant group differences. By using multivariate logistic regression, the investigation looked into the connection between SUA and both diabetes and prediabetes. The results from the multivariate logistic regression model are presented as odds ratios (ORs) and 95% confidence intervals (CIs). Model 1 was unadjusted data. Model 2 was adjusted for age, sex, smoking, drinking, hypertension, hyperlipidemia, BMI, WC, SBP, and DBP. Then, age, sex, smoking, drinking, hypertension, hyperlipidemia, BMI, WC, SBP, DBP, TG, TC, HDL-C, LDL-C, BUN, and Cr were adjusted to build model 3. In addition, the study evaluated the dose–response effect between SUA levels and both diabetes and prediabetes in sexes utilizing a restricted cubic spline (RCS) regression model. The level of statistical significance was set at *P* < .05.

## 3. Results

### 3.1. Baseline characteristics of the participants

There were 190,891 participants, including 106,482 men and 84,409 women. The mean age of the study individuals was 43.10 ± 12.18 years. The mean SUA level was 351.12 ± 91.57 μmol/L, including 401.63 ± 79.39 μmol/L for males and 287.39 ± 61.09 μmol/L for females. There were 46,240 (24.2% of total) patients with prediabetes, including 28,791 (27.0%) males and 17,449 (20.7%) females. Those with diabetes numbered 20,792 (10.9% of total), of which 13,175 (12.4%) were males and 7617 (9.0%) were females. The baseline characteristics of the SUA quartile groups and of all subjects are summarized in Table 1. In contrast to the subjects in the SUA Q1 group, the subjects in the SUA Q4 group were likely to be males, older, current smokers, and current drinkers, had a greater percentage of hypertension and hyperlipidemia, had a greater percentage of prediabetes and diabetes, had higher levels of BMI, WC, SBP, DBP, FPG, HbA1_C_, TC, TG, LDL-C, BUN, and Cr, and had lower levels of HDL-C. All variables were considered statistically significant in the SUA groups.

**Table 1:** The baseline characteristics of the serum uric acid quartile groups and of all subjects.

Variable	Overall	Quartiles of serum uric acid				F/H/χ^2^	*P* value
	(n = 190891)	Q1 (n = 48231)	Q2 (n = 47738)	Q3(n = 47367)	Q4 (n = 47555)		
Serum uric acid, μmol/L	351.12 ± 91.57	242.28 ± 29.93	313.51 ± 17.89	376.82 ± 19.21	473.65 ± 54.37	*F* = 354880.604	<.001
Gender, N (%)							
Males	106,482 (55.8%)	5343 (11.1%)	19,726 (41.3%)	36,722(77.5%)	44,691(94.0%)	χ^2^ = 80331.684	<.001
Females	84,409 (44.2%)	42,888 (88.9%)	28,012 (58.7%)	10,645(22.5%)	2864 (6.0%)		
Age, yr	43.10 ± 12.18	42.12 ± 11.52	43.72 ± 12.54	43.88 ± 12.35	42.68 ± 12.19	*F* = 237.200	<.001
BMI, kg/m^2^	23.70 ± 3.62	22.06 ± 3.68	23.10 ± 3.36	24.23 ± 3.17	25.45 ± 3.31	*F* = 8510.646	<.001
WC, cm	82.20 ± 10.66	74.73 ± 8.61	79.71 ± 9.74	85.08 ± 9.24	89.40 ± 8.79	*F* = 25313.374	<.001
SBP, mm Hg	121.41 ± 16.52	115.80 ± 16.32	119.98 ± 16.71	123.58 ± 15.83	126.39 ± 15.25	*F* = 3994.703	<.001
DBP, mm Hg	72.82 ± 11.11	68.78 ± 10.28	71.52 ± 10.72	74.31 ± 10.82	76.74 ± 10.98	*F* = 5019.587	<.001
FPG, mmol/L	5.29 ± 1.11	5.15 ± 1.09	5.29 ± 1.19	5.35 ± 1.12	5.38 ± 1.01	*F* = 450.195	<.001
HbA1_C_, %	5.46 ± 0.73	5.38 ± 0.73	5.47 ± 0.78	5.49 ± 0.73	5.50 ± 0.66	*F* = 267.931	<.001
TC, mmol/L	4.88 ± 0.96	4.71 ± 0.91	4.84 ± 0.95	4.90 ± 0.96	5.06 ± 0.98	*F* = 1195.576	<.001
TG, mmol/L	1.22 (0.99)	0.90 (0.56)	1.09 (0.78)	1.37 (0.99)	1.76 (1.34)	*H* = 35014.009	<.001
HDL-C, mmol/L	1.28 ± 0.32	1.45 ± 0.32	1.33 ± 0.32	1.21 ± 0.29	1.13 ± 0.26	*F* = 10871.115	<.001
LDL-C, mmol/L	2.78 ± 0.78	2.58 ± 0.74	2.80 ± 0.77	2.89 ± 0.78	2.98 ± 0.81	*F* = 1576.931	<.001
BUN, mmol/L	4.70 (1.60)	4.31 (1.56)	4.64 (1.59)	4.85 (1.55)	4.98 (1.55)	*H* = 8040.454	<.001
Cr, µmol/L	70.00 (23.00)	57.00 (11.00)	65.00 (19.00)	76.00 (18.00)	82.00 (16.00)	*H* = 70832.111	<.001
Smoking, N (%)	15,611 (8.2%)	1029 (2.1%)	2945 (6.2%)	5055 (10.7%)	6582 (13.8%)	χ^2^ = 5026.418	<.001
Drinking, N (%)	20,664 (10.8%)	1909 (4.0%)	3883 (8.1%)	6348 (13.4%)	8524 (17.9%)	χ^2^ = 5522.983	<.001
Past history, N (%)							
Hypertension	52,734 (27.6%)	11,489 (23.8%)	12,820 (26.9%)	13,424(28.3%)	15,001(31.5%)	χ^2^ = 740.799	<.001
Hyperlipoidemia	13,714 (7.2%)	1765 (3.7%)	2754 (5.8%)	3876 (8.2%)	5319 (11.2%)	χ^2^ = 2254.363	<.001
Glucose metabolism state, N (%)							
Normoglycaemia	123,859 (64.9%)	35,225 (73.0%)	31,455 (65.9%)	29,492(62.3%)	27,687(58.2%)	χ^2^ = 2496.727	<.001
Prediabetes	46,240 (24.2%)	8768 (18.2%)	10,825 (22.7%)	12,368(26.1%)	14,279(30.0%)	χ^2^ = 1986.558	<.001
Diabetes	20,792 (10.9%)	4238 (8.8%)	5458 (11.4%)	5507 (11.6%)	5589 (11.8%)	χ^2^ = 297.234	<.001

The quartiles of serum uric acid were calculated respectively (Q1: ≤282 μmol/L, Q2: 282–344 μmol/L, Q3: 344–411 μmol/L, and Q4: >411 μmol/L).

BMI = body mass index, BUN = blood urea nitrogen, Cr = creatinine, DBP = diastolic blood pressure, FPG = fasting plasma glucose, HbA1_C_ = glycosylated haemoglobin, HDL-C = high-density lipoprotein cholesterol, LDL-C = low-density lipoprotein cholesterol, SBP = systolic blood pressure, TC = total cholesterol, TG = triglyceride, WC = waist circumference.

Data are presented as the mean ± SD, median (interquartile range, IQR), and prevalence.

*F*, ANOVA; *H*, Kruskal–Wallis *H* test; χ^2^, chi-square test.

### 3.2. SUA levels and prediabetes and diabetes

By using multivariate logistic regression, the relationship between prediabetes and SUA levels is showed in Table 2 and the association between diabetes and SUA levels is displayed in Table 3. With or without adjustment for confounders, a higher level of SUA had a positive association with prediabetes in all subjects. In Model 3, compared to quartile 1 of SUA, the prevalence of prediabetes increased in quartile 4 of SUA (OR = 1.378, 95% CI = 1.321–1.437, *P* for trend < .001). However, a higher level of SUA had a significant negative association with diabetes among all participants. The prevalence of diabetes decreased in SUA quartile 4 when compared to quartile 1 (OR = 0.690, 95% CI = 0.651–0.730, *P* for trend < .001).

**Table 2:** Odds ratios for prediabetes according to serum uric acid groups in various models.

SUA, μmol/L	Prediabetes OR (95% CI)		
	Model 1	Model 2	Model 3
*All*			
Per 1 increment	1.003 (1.002–1.003)***	1.002 (1.001–1.002)***	1.001 (1.001–1.002)***
Quartile			
Q1 (≤282)	Reference	Reference	Reference
Q2 (282–344)	1.320 (1.279–1.362)***	1.128 (1.090–1.167)***	1.094 (1.057–1.132)***
Q3 (344–411)	1.590 (1.542–1.640)***	1.257 (1.211–1.306)***	1.202 (1.157–1.249)***
Q4 (>411)	1.931 (1.874–1.991)***	1.474 (1.416–1.535)***	1.378 (1.321–1.437)***
*P* for trend	<.001	<.001	<.001
*Males*			
Per 1 increment	1.002 (1.002–1.002)***	1.002 (1.001–1.002)***	1.001 (1.001–1.001)***
Quartile			
Q1 (≤348)	Reference	Reference	Reference
Q2 (348–396)	1.088 (1.046–1.132)***	1.115 (1.071–1.161)***	1.114 (1.069–1.161)***
Q3 (396–450)	1.218 (1.172–1.266)***	1.222 (1.173–1.272)***	1.209 (1.161–1.260)***
Q4 (>450)	1.410 (1.357–1.465)***	1.359 (1.304–1.416)***	1.328 (1.272–1.386)***
* P* for trend	<.001	<.001	<.001
*Females*			
Per 1 increment	1.004 (1.004–1.004)***	1.001 (1.001–1.002)***	1.001 (1.001–1.001)***
Quartile			
Q1 (≤246)	Reference	Reference	Reference
Q2 (246–281)	1.085 (1.032–1.141)***	1.023 (0.971–1.078)	1.004 (0.953–1.059)
Q3 (281–322)	1.290 (1.228–1.354)***	1.114 (1.058–1.173)***	1.068 (1.014–1.126)*
Q4 (>322)	1.824 (1.741–1.912)***	1.265 (1.202–1.332)***	1.184 (1.122–1.249)***
* P* for trend	<.001	<.001	<.001
*P* for interaction	<.001	<.001	<.001

95% CI = 95% confident interval, OR = odds ratio, SUA = serum uric acid.

* *P* < .05.

*** *P* < .001.

**Table 3:** Odds ratios for diabetes according to serum uric acid groups in various models.

SUA, μmol/L	Diabetes OR (95% CI)		
	Model 1	Model 2	Model 3
*All*			
Per 1 increment	1.001 (1.001–1.001)***	0.998 (0.998–0.998)***	0.998 (0.998–0.999)***
Quartile			
Q1 (≤282)	Reference	Reference	Reference
Q2 (282–344)	1.340 (1.285–1.398)***	0.994 (0.949–1.040)	0.996 (0.951–1.043)
Q3 (344–411)	1.366 (1.309–1.425)***	0.802 (0.762–0.843)***	0.809 (0.768–0.852)***
Q4 (>411)	1.382 (1.325–1.442)***	0.672 (0.636–0.710)***	0.690 (0.651–0.730)***
*P* for trend	<.001	<.001	<.001
*Males*			
Per 1 increment	0.998 (0.998–0.998)***	0.997 (0.997–0.997)***	0.997 (0.997–0.997)***
Quartile			
Q1 (≤348)	Reference	Reference	Reference
Q2 (348–396)	0.677 (0.644–0.712)***	0.650 (0.617–0.686)***	0.658 (0.624–0.695)***
Q3 (396–450)	0.674 (0.641–0.709)***	0.593 (0.562–0.625)***	0.602 (0.570–0.636)***
Q4 (>450)	0.693 (0.659–0.728)***	0.537 (0.508–0.567)***	0.552 (0.521–0.585)***
* P* for trend	<.001	<.001	<.001
*Females*			
Per 1 increment	1.005 (1.004–1.005)***	1.002 (1.002–1.002)***	1.002 (1.001–1.002)***
Quartile			
Q1 (≤246)	Reference	Reference	Reference
Q2 (246–281)	1.071 (0.995–1.153)	1.005 (0.932–1.084)	1.005 (0.932–1.085)
Q3 (281–322)	1.289 (1.201–1.384)***	1.097 (1.020–1.180)*	1.076 (0.999–1.160)
Q4 (>322)	1.958 (1.832–2.091)***	1.321 (1.231–1.419)***	1.282 (1.190–1.381)***
* P* for trend	<.001	<.001	<.001
*P* for interaction	<.001	<.001	<.001

95% CI = 95% confident interval, OR = odds ratio, SUA = serum uric acid.

* *P* < .05.

*** *P* < .001.

### 3.3. Subgroup analyses

**Figure 1.**
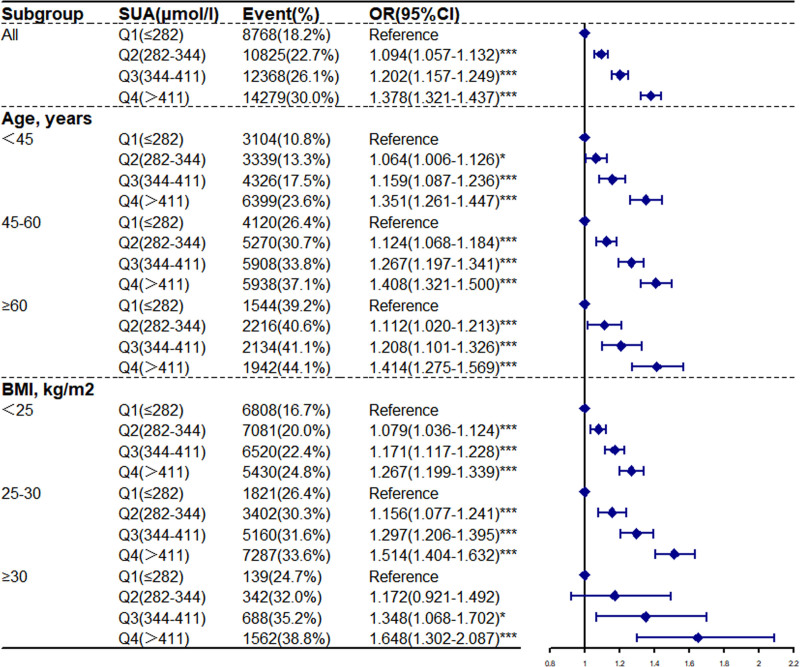
Adjusted odd ratios for prediabetes with quartiles of serum uric acid. According to baseline characteristics, analyses were using model 3. * *P* < .05, ** *P* < .01, *** *P* < .001.

**Figure 2.**
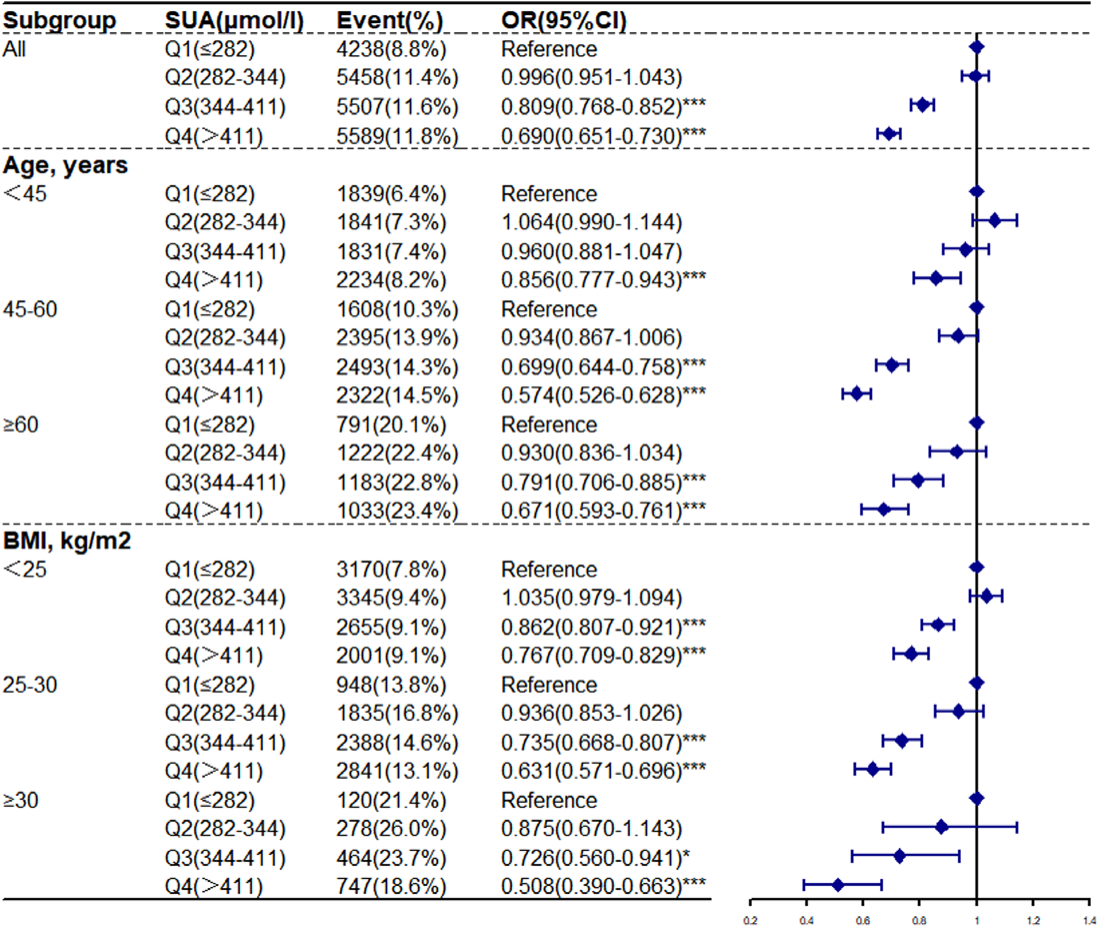
Adjusted odd ratios for diabetes with quartiles of serum uric acid. According to baseline characteristics, analyses were using model 3. * *P* < .05, ** *P* < .01, *** *P* < .001.

In Figures 1 and 2, logistic regression analysis was performed by grouping all subjects according to different age and BMI groups. The cutoff of age was <45, 45 to 60, and ≥60 years. The cutoffs for BMI were <25, 25 to 30, and ≥30 kg/m^2^. Using the Q1 groups as the reference, the odds ratios (ORs) of prediabetes in the Q4 groups increased among different age groups (OR = 1.351, 95% CI = 1.261–1.447) (OR = 1.408, 95% CI = 1.321–1.500) (OR = 1.414, 95% CI = 1.275–1.569) and different BMI groups (OR = 1.267, 95% CI = 1.199–1.339) (OR = 1.514, 95% CI = 1.404–1.632) (OR = 1.648, 95% CI = 1.302–2.087). However, the diabetes ORs of the participants in the Q4 groups decreased among different age groups (OR = 0.856, 95% CI = 0.777–0.943) (OR = 0.574 95% CI = 0.526–0.628) (OR = 0.671, 95% CI = 0.593–0.761) and different BMI groups (OR = 0.767, 95% CI = 0.709–0.829) (OR = 0.631, 95% CI = 0.571–0.696) (OR = 0.508 95% CI = 0.390–0.663). We found that elevated SUA had a positive correlation with a high prevalence of prediabetes and a negative correlation with diabetes among various age and BMI groups. The findings of multivariate regression analysis among age and BMI groups were consistent with those of all subjects.

## 4. Gender differences

**Figure 3.**
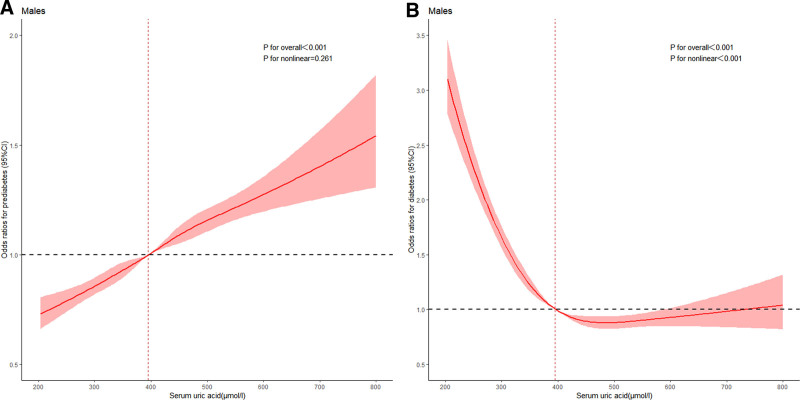
Dose–response relationship between serum uric acid and prediabetes in males (A) and serum uric acid and diabetes in males (B). The adjustments were age, smoking, drinking, hypertension, hyperlipidemia, BMI, WC, SBP, DBP, TG, TC, HDL-C, LDL-C, BUN, and Cr. BMI = body mass index, BUN = blood urea nitrogen, Cr = creatinine, DBP = diastolic blood pressure, HDL-C = high-density lipoprotein cholesterol, LDL-C = low-density lipoprotein cholesterol, SBP = systolic blood pressure, TC = total cholesterol, TG = triglyceride.

**Figure 4.**
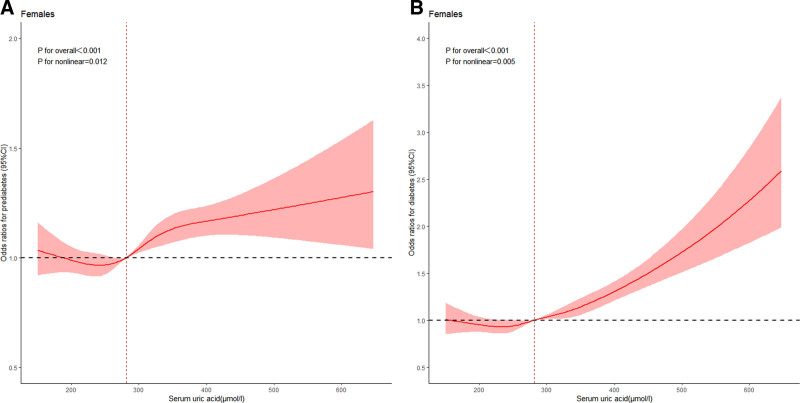
Dose–response relationship between serum uric acid and prediabetes in females (A) and serum uric acid and diabetes in females (B). The adjustments were age, smoking, drinking, hypertension, hyperlipidemia, BMI, WC, SBP, DBP, TG, TC, HDL-C, LDL-C, BUN, and Cr. BMI = body mass index, BUN = blood urea nitrogen, Cr = creatinine, DBP = diastolic blood pressure, HDL-C = high-density lipoprotein cholesterol, LDL-C = low-density lipoprotein cholesterol, SBP = systolic blood pressure, TC = total cholesterol, TG = triglyceride.

The quartiles of the SUA concentration were calculated by sex separately in Tables 2 and 3. The cutoff values for the uric acid concentration were ≤246, 246 to 281, 281 to 322, and >322 μmol/L in females. In males, the cutoff values for uric acid concentration were ≤348, 348 to 396, 396 to 450, and >450 μmol/L. In comparison to the reference group, the ORs of prediabetes in the Q4 groups increased among females (OR = 1.184, 95% CI = 1.122–1.249, *P* for trend < .001), and the ORs of diabetes in the Q4 groups also increased among females (OR = 1.282, 95% CI = 1.190–1.381, *P* for trend < .001). In males, the ORs of prediabetes in the Q4 groups increased (OR = 1.328, 95% CI = 1.272–1.386, *P* for trend < .001) compared with the reference group. The risk of prediabetes was more significant in men than in women (*P* for interaction < .001). However, we found that the ORs of diabetes in the Q4 groups decreased in males (OR = 0.552, 95% CI = 0.521–0.585, *P* for trend < .001). Dose–response relationship between SUA and prediabetes and diabetes analyzed by taking a 5-node restricted cubic spline (RCS) curve among females and males in Figures 3 and 4. The findings from the multivariate regression analysis were similar to the findings of the RCS curve.

## 5. Discussion

An overall sample size of 190,891 participants was used in this cross-sectional study, and it discovered that elevated SUA levels were positively associated with prediabetes and negatively associated with diabetes. Further analysis revealed a significantly positive correlation between SUA levels and prediabetes in both sexes. Excessive SUA decreased the incidence of diabetes in males but increased the incidence of diabetes in females.

The link between SUA and the incidence of prediabetes has been evaluated by some studies. Some studies found a notable connection between SUA and prediabetes, with a positive correlation. Hairong Nan and colleagues, in a study of 3632 subjects from Qingdao, China, found that SUA levels in nondiabetic patients increased with increasing FPG levels.^[25]^ A study by Ryoung Jin Park and colleagues in 4633 subjects found that higher SUA levels were linked to increased prediabetes in Korea.^[30]^ However, others showed a negative association. Tangigul Haque and colleagues found in a study of 310 Bangladeshi subjects that uric acid levels were increased in healthy individuals and decreased in the prediabetic population.^[24]^ The discrepancy may be the result of racial differences as well as the small sample size in the study population. In this study, our findings were that elevated SUA was positively correlated with prediabetes. Insulin resistance could be considered to be a potential underlying mechanism in prediabetes.^[31]^ Higher uric acid levels can lead to an inflammatory response in pancreatic islet cells, oxidative stress, and increased levels of tumor necrosis factor-α, reducing nitric oxide bioavailability, leading to endothelial dysfunction, further inducing insulin resistance and promoting the development of prediabetes.^[10,32,33]^ In addition, higher insulin levels further reduce uric acid excretion in the kidneys.^[29]^

This study further compared the differences between SUA and prediabetes and diabetes. Using the Q1 groups as the reference, the ORs of prediabetes in the Q4 groups increased 37.8%. However, the ORs of diabetes in the Q4 groups decreased 31.0%. In addition, we analyzed age and BMI groups and found similar results: elevated SUA had a positive correlation with a high prevalence of prediabetes and a negative correlation with diabetes. Previous studies found a positive association between hyperuricemia and the risk of diabetes. The results we found are inconsistent with these studies. A study found that prediabetic individuals had a higher prevalence of gout, while diabetic individuals had a lower prevalence of gout than prediabetic individuals.^[34]^ This result is consistent with our findings. The probable mechanism is that the increased uric acid excretion of glycosuria plays a major role, rather than the decrease in uric acid production. When blood glucose levels are elevated beyond the maximum reabsorption capacity of the kidney, glucose is excreted into the urine. High glucose levels in the urine further accelerate glucose transport by glucose transporter 9, leading to an increase in uric acid exchange in the renal tubular apical membrane.^[24,35,36]^ In renal proximal tubules, high glucose levels in the urine can also competitively inhibit the reabsorption of uric acid. As a consequence, SUA levels decrease.^[24,26]^

Studies on sex-specific associations between SUA and prediabetes have been controversial. A study revealed that high SUA levels were positively associated with the risk of prediabetes in the Japanese population and were significant in women but no men.^[37]^ A study of a Dutch population reported that increased SUA levels were linked to the emergence of prediabetes in normoglycaemic females, but this association was not observed in normoglycaemic males.^[30]^ A study of North China found that a higher risk of impaired fasting glucose was associated with SUA concentrations in men but not women.^[38]^ This cross-sectional analysis found a positive association between SUA and prediabetes that was more significant in males than in females. However, in the association between SUA and diabetes, there was a positive correlation in women and the opposite in men. The mechanism behind these sex differences remains unclear, and it is possible that estrogen plays a role.^[39]^ It has been supported that lower estrogen levels in men may be linked to higher insulin resistance.^[39]^ Further research is needed to verify the mechanisms of gender differences in the association.

This study had a significant advantage due to its large sample size, which comparatively thorough investigated the association between SUA levels and the prevalence of prediabetes and diabetes in all participants and among the different sexes, providing theoretical help for the early recognition of prediabetes in a normal Chinese population. However, this study still had some shortcomings. First, this study was a cross-sectional study, and no causal conclusions could be drawn. Second, postprandial plasma glucose has not been widely utilized in the normal population. Finally, because the study population was mostly composed of individuals from Zhejiang Province in China, the conclusions drawn from the findings may not necessarily apply to other countries. In order to delay or even control the progression to diabetes, early identification of prediabetes and lifestyle modifications are of great importance. In the future, there is a need for large-scale prospective cohort validation studies.

## 6. Conclusion

The study involved 190,891 participants and showed that a higher level of SUA was strongly associated with an increased prevalence of prediabetes and associated with a decreased prevalence of diabetes. Moreover, the results indicated that males with elevated SUA levels showed a higher prediabetes prevalence than females. Therefore, more attention should be paid to SUA levels when screening for prediabetes in a normal Chinese population, especially in males. It may contribute to the early identification of prediabetic states and the timely provision of targeted health advice for the early prevention of diabetes. To validate this finding, a large prospective study will be conducted in the future.

## Author contributions

**Conceptualization:** Liying Chen.

**Data curation:** Yilin Huang, Junlu Zhang, Liangli Chen.

**Formal analysis:** Keqing Shen.

**Funding acquisition:** Liying Chen.

**Investigation:** Keqing Shen.

**Methodology:** Keqing Shen.

**Software:** Keqing Shen, Jingyi Li, Lusha Li.

**Validation:** Keqing Shen.

**Writing – original draft:** Keqing Shen.

**Writing – review & editing:** Xixuan Cai, Jianjiang Pan, Liying Chen.
